# Genome analysis revealed novel genotypes and recombination of the human parechoviruses prevalent in children in Eastern China

**DOI:** 10.1186/s13099-016-0135-z

**Published:** 2016-11-08

**Authors:** Xiangyang Zhao, Yongqiang Shi, Yu Xia

**Affiliations:** Nanjing Lishui People’s Hospital, Lishui, Jiangsu 211200 China

**Keywords:** Human parechovirus, Genome sequence, Phylogenetic analysis, Recombination

## Abstract

**Background:**

Human parechovirus (HPeV) is a genus of virus in the family *Picornaviridae*, having two species A and B. HPeVs are common infectious agents, usually causing mild diarrhea and respiratory disease in young children.

**Results:**

Here, we collected and sequenced the near complete genome sequences of 17 novel HPeVs from children with diarrhea in eastern China, which showed significant nucleotide sequence divergence. Phylogenetic analysis based on the complete genomes of these HPeV strains revealed that they belonged to seven different genotypes (including three putative novel genotypes), suggesting that HPeVs showed genotype diversity in this area. Recombination analysis indicated that one of the HPeVs, belonging to HPeV-1 with strain name 146Chzj02, was a recombinant generated by inter-genotype recombination among three HPeV strains belonging to three different genotypes, respectively.

**Conclusion:**

Our data revealed the property of the genotype diversity of HPeVs prevalent in children with diarrhea in eastern China, which will be helpful in the future study of the viral evolution of HPeVs and the identification and typing of HPeVs in the clinical laboratory.

## Background

Human parechoviruses (HPeVs) belong to Parechovirus genus in the *Picornaviridae* family, which are non-enveloped, positive-sense RNA viruses with icosahedral capsids [1]. HpeVs have a genome of about 7300nt which flanked by an un-translated region (UTR) at both the 5′ and 3′ ends. The gnome encodes a polyprotein which is post-translationally cleaved by virus proteases to produce the structural (VP0, VP3 and VP1) and non-structural (2A–C and 3A–D) proteins [2].

HPeVs are frequent infectious agents, usually causing mild gastroenteritis and respiratory disease in young children, however, more serious cases, such as flaccid paralysis, encephalitis and myocarditis, have also been reported. HPeV is subdivided into 16 different genotypes [3–5]. The prototypes of HPeVs, including HPeV-1 and HPeV-2, were originally classified as echo-22 and echo-23, in enterovirus but were regrouped as a separate genus according to their genome organization and biological properties [6, 7]. Among these HPeV genotypes, some were associated with certain specific diseases. HPeV-1 is widely prevalent throughout the world and often found in children with diarrhea and gastroenteritis [3, 8]. HPeV-3 was particularly associated with sepsis syndromes and aseptic meningitis/meningoencephalitis in children [9, 10]. HPeV-4 was isolated from a 5 year old patient with lymphadenitis [11] and might also cause neonatal sepsis [12]. HPeV-6 was discovered from an infant with Reye syndrome [13]. HPeV-7 and HPeV-8 were identified from a patient with non-polio acute flaccid paralysis and a patient with enteritis, respectively [14]. HPeV-10 and -11 were identified in patients with acute gastroenteritis [15, 16]. HPeV-12 was detected in an infant with diarrhea and paralysis [17] while HPeV-14 was discovered from a febrile child [18].

In the present study, we sequenced 17 HPeVs genome sequences from the fecal samples from children <6 years of age with diarrhea, finding three putative new genotypes. Recombination analysis was also performed based on these genomes and one putative recombinant was found.

## Methods

### Samples and HPeVs detection

From Jan. 2011 to Jun. 2014, a total of 634 stool specimens which were collected from children under 6 years of age with diarrhea who were treated as outpatients were subjected to RT-PCR assay, where the degenerate primers were designed to amplify the *VP3/VP1* region of HPeVs, which produced ~360 bp specific bands [19]. The positive PCR products were sequenced and then subjected to BLASTn in GenBank to find whether the HPeV strains belong to putative novel genotypes, where the sequences with <85% similarity to the best BLASTn matches in GenBank were considered to be putative novel genotypes and selected for genome sequencing.

### Genome sequencing

RT-PCRs were used to acquire the genome. Primers were designed based on the complete genomes in GenBank which showed high similarities to the 360 bp sequences got in the RT-PCR screening in this study. Briefly, the total RNA was extracted from 200 μl fecal supernatants using QIAamp Viral RNA Mini Kit (Qiagen, Germany) in accordance with the manufacturer’s protocol. RT-PCR was performed by using TaKaRa RNA PCR kit (TaKaRa, Japan). The parameters of PCR amplification included an initial incubation at 94 °C for 5 min, followed by 30 cycles of denaturation at 94 °C for 40 s, annealing for 40 s at a temperature varied depending on different primers, and extension at 72 °C for 1 min, with a final incubation at 72 °C for 10 min. The specific bands were excised from the gel and sequenced by Sanger method (Shanghai Sangon, China).

### Phylogenetic analysis

To investigate the relationship between the HPeVs in this study and those with complete genomes in GenBank, all the HPeV genomes were retirved from GenBank and phylogenetic analysis was performed based on the complete genomes. The HPeVs genome sequences from GenBank were pre-analyzed and those sequences showing less than 1% divergence from each other were considered as the same strain and only one of them were included in the phylogenetic analysis. Sequence alignment was performed using CLUSTAL W with the default settings [20]. A phylogenetic tree with 1000 bootstrap resamples of the alignment data sets was generated using the neighbor-joining method in MEGA5.0 [21]. Sequence divergence based on the complete genome of HPeVs was analyzed using AliGROOVE software [22]. The scores were ranging from −1, indicating full random similarity, to +1, non-random similarity.

### Virus recombination detection

Detection of potential recombinant sequences, identification of potential parental sequences, and localization of possible recombination break points were determined using the Recombination Detection Program (RDP3.0) [23]. The potentially significant recombination events were further confirmed by SimPlot software [24] and phylogenetic analysis based on the exchanged sequences of the recombinants.

## Results

### Phylogenetic characterization of the novel HPeVs

**Fig. 1:**
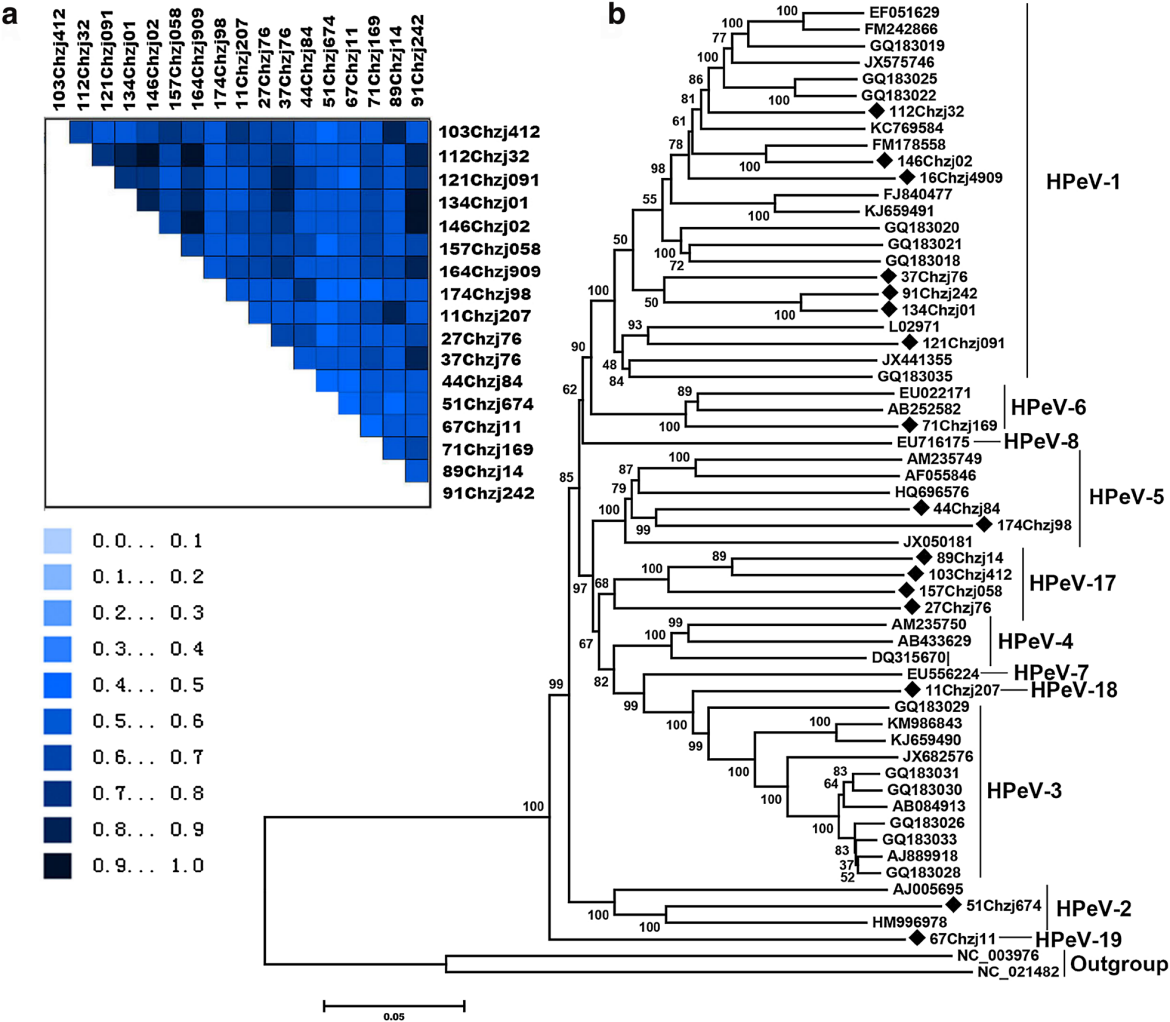
Sequence divergence and phylogenetic analysis based on the complete genome of HPeVs in the present study. **a** Nucleotide sequence divergence of the 17 HPeVs in this study was established using AliGROOVE software. The scores were ranging from 0 to +1 indicating the non-random similarity. The *darker blue* indicated the higher non-randomized accordancy between pairwise sequence comparisons. Strain names in this study were listed on *top* and the *right hand side* of the matrix; **b** neighbor joining phylogenetic tree was constructed with Mega5.0 from multiple alignments of the complete genome of the 17 HPeVs in the present study and other 47 HPeVs with complete genome sequence available in GenBank. Two Ljungan virus strains were included as outgroup. *Bootstrap values* are indicated at the *nodes*. The *scale bar* indicates the number of substitutions per position for a unit branch length. Viruses identified in the present study were displayed with diamonds

RT-PCR screening indicated 83 (13.1%) of the total 634 stool specimens were positive for HPeVs genes. Sequence analysis indicated that 17 sequences showed <85 % similarity to the previous HPeV strains in GenBank, which were then selected for further genome sequencing. In order to characterize these strains, the nearly complete genomes of the 17 putative novel HPeV strains were sequenced and submitted to GenBank with GenBank nos. KT879915–KT879931. The heterogeneity of sequence divergence based on the complete genomes of the 17 HPeVs was indicated in Fig. 1a, which showed that the 17 shared 71–88% identities among themselves, suggesting these HPeVs showed the property genotype diversity. Phylogenetic analysis based on the complete genome of the 17 HPeVs in the present study and those HPeVs with complete genome available in GenBank indicated that the 17 HPeVs identified in this study belonged to 7 different genotypes (Fig. 1b). Seven of them belonged to HPeV-1, being grouped into 4 different clusters within the HPeV-1 clade. One strain clustered with an HPeV-2 strain (HM996978), sharing 82.2% sequence similarity. Two strains clustered together within the HPeV-5 clade, sharing 80.1% sequence identity with each other. One strain belonged to HPeV-6, clustering and sharing 85% sequence identity the other two previous HPeV-6 strains in the same clade. Five of the strains clustered together into a separate group with 74–78% sequence similarities with all the other HPeVs, suggesting they belonged to a new genotype (HPeV-17). One strain clusters separately, sharing 75–83% sequence similarities to all the other HPeVs, representing a new genotype (HPeV-18). The rest one strain formed the deepest branch, sharing 73–77% sequence identities with all the other HPeVs strains, and belonged to a putative new genotype, HPeV-19.

### Recombination of HPeVs

**Fig. 2:**
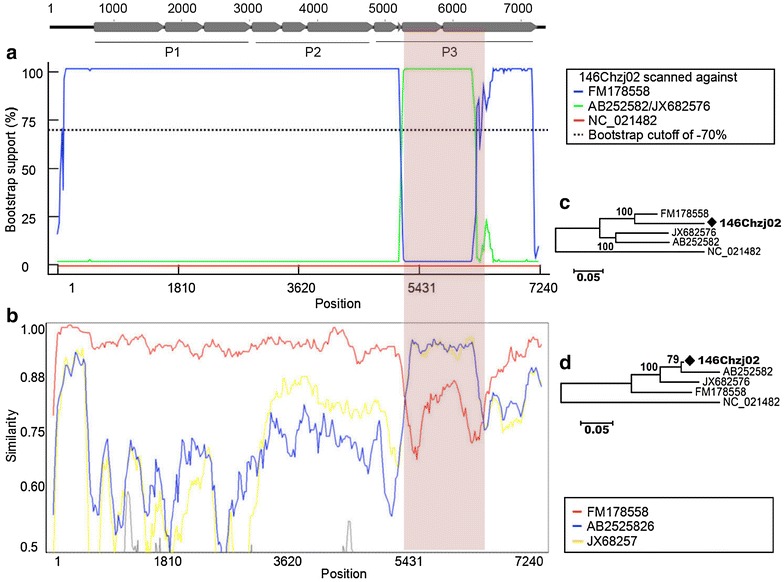
Identification of the recombination event based on complete genome of the potential recombinant 146Chzj02. **a** BOOTSCAN evidence for the recombination origin on the basis of pairwise distance, modeled with a window size 200, step size 20, and 100 Bootstrap replicates using RDP3.0 software; **b** similarity analysis of complete genomes of potential recombinant 146Chzj02 and its three potential parental strains using Simplot Version 3.5.1 software by a sliding window of 200 nucleotides moving in steps of 20 nucleotides. The shadowy area indicated the recombination event. **c** Neighbor joining tree constructed using the recombinant region (5694–6798nt); **d** neighbor joining tree constructed using the non-recombinant region. The nucleotide position is indicated based on the genome sequence of 146Chzj02

One potentially significant recombination event was found with a high degree of confidence. Figure 2a, b indicated the bootscan and similarity plots, respectively, of the recombination event which belonged to an intra-genotype recombination occurred between the major parent of an HPeV-1 strain (FM178558) and two minor parents including an HPeV-6 strain (AB252582) and an HPeV-3 strain (JX682576), leading to the recombinant 146Chzj02. Figure 1a, b displayed that this recombination event occurred within the P3 gene of HPeVs. Among the four strains related with this recombination event, FM178558 belongs to HPeV-1 which was isolated from human feces from Netherland [25]. AB252582 was isolated from cerebrospinal fluid from a patient in Japan [13], while JX682576 was isolated from feces of a newborn with gastroenteritis in Germany [26]. To confirm the recombination event, the relevant strains were analyzed by neighbor joining trees using MEGA5.0. Figure 2c, d indicated the trees constructed on the recombinant region and the non-recombinant regions, respectively. The recombinant strain 146Chzj02 clustered closely with its major and minor parental strains in the two phylogenetic trees, respectively. Taken together, our data clearly indicated the existence of the putative recombination event among AB252582, JX682576, and FM178558, which belonged to an inter-genotype recombination.

To verify the whether recombinant is naturally present in the fecal sample, two sets of specific primers were designed basing on the genome sequence of 146Chzj02, which amplified two fragments including the two recombination breakpoints, respectively. PCR amplified specific bands were sequenced and sequencing results of the two types of PCR amplicons were identical to the originally determined genome, suggesting that 146Chzj02 is natural recombinant.

## Discussion

Although many reports indicated that HPeVs can cause infection in young children and lead to various diseases including diarrhea, respiratory diseases, encephalomyelitis, meningitis, myocarditis, lymphadenopathy, hemolytic uremic syndrome, and sudden infant death syndrome, no causal relationship has been established except with HPeV-3 being primarily associated with neonatal sepsis [27, 28]. In the present study, we sequenced the complete genomes of 17 HPeV strains from children <6 years old with diarrhea in eastern China. Up to now, there are totally 16 different types of HPeV, of which type 6 to 16 were identified as genotype by genetic analysis based on sequence divergence [6, 7]. However, we did not include a control group in our study, which provide no sufficient proof of HPeV association to the diarrhea of these children included in this study. Besides, mere detection of HPeV in clinical samples does not confirm or establish its role in diarrhea, as even the asymptomatic individuals may continue to shed HPeVs for longer periods of time [29]. Phylogenetic analysis based on the complete genome sequences of 17 HPeV strains indicated they belonged to 7 different types, which suggested that HPeVs in eastern China showed property of genotype diversity. HPeV1 is the most frequently detected type worldwide, which is also the major genotype in the present study. Considering such a vast diversity of HPeVs prevalent in children population in eastern China, a highly sensitive RT-PCR assay should be designed covering more genotypes of HPeVs in laboratory diagnosis.

Recombination is a rather common phenomenon in picornaviruses [30] and understanding recombination will be helpful in study of the evolution of pathogens and the development of viral vaccine. Like other picornaviruses, recombination in HPeVs has also been found, with breaking points at the capsid encoding and non-structural encoding regions [31–34], among these recombination events most belonged to intra-genotype recombination. In the present study, a recombination event was detected in the non-structural region, which was inter-genotype recombination occurred between 3 HPeV strains belong three different genotypes and led to an HPeV-1 recombinant. This finding would support that the most frequent breaking points for recombination in HPeVs flanked the capsid-encoding region [2, 3, 25]. Our data indicated that the recombinant (146Chzj02) and its three parental strains (including AB252582, JX682576, and FM178558) were from four different countries, respectively, which implies that this recombination event might have occurred among the ancestors of the three parental HPeV strains long time ago, when they were simultaneously circulated in a single area in that meantime.

## Conclusion

We sequenced 17 complete genome sequences of HPeV from the feces of Children <6 years old with diarrhea in eastern China, which belonged to seven different genotypes including three putative novel genotypes based on phylogenetic analysis. Recombination analysis indicated that one of the HPeVs in the current study belonged to putative recombinant produced by inter-genotype recombination occurred among 3 different genotypes of HPeVs.

Our data provided the genetic diversity of HPeVs from children with diarrhea in eastern China, which will be helpful in the future study of the viral evolution of HPeVs and the identification and typing of HPeVs in the clinical laboratory.

## Abbreviations

HPeV: human parechovirus
PCR: polymerase chain reaction
UTR: un-translated region
